# Rio Mamore Virus and Hantavirus Pulmonary Syndrome, Brazil 

**DOI:** 10.3201/eid2009.131472

**Published:** 2014-09

**Authors:** Renata Carvalho de Oliveira, Marcelo Cordeiro-Santos, Alexandro Guterres, Jorlan Fernandes, Alexsandro X. de Melo, Guilherme A.P. João, Maria A.M. Novais, Elizabeth Salbé Travassos da Rosa, Pedro Fernando da Costa Vasconcelos, Stefan Vilges de Oliveira, Bernardino Cláudio de Albuquerque, Elba Regina Sampaio de Lemos

**Affiliations:** Oswaldo Cruz Foundation, Rio de Janeiro, Brazil (R.C. Oliveira, A. Guterres, J. Fernandes, E.R.S. Lemos);; Health Surveillance Foundation, Manaus, Brazil (M. Cordeiro-Santos, A.X. de Melo, M.A.M. Novais, B.C. Albuquerque);; Amazon State University, Manaus (M. Cordeiro-Santos); Tropical Medicine Foundation, Manaus (G.A.P. João);

**Keywords:** Hantavirus, Rio Mamore virus, hantavirus pulmonary syndrome, Brazilian Amazon, viruses

**To the Editor**: Hantavirus pulmonary syndrome (HPS) is an acute, severe, frequently fatal disease associated with cardiopulmonary failure; it is caused by hantaviruses naturally hosted by wild rodents. Rio Mamore virus (RIOMV) was first described in 1996 in Bolivia; it was associated with the small-eared pygmy rice rat, *Oligoryzomys microtis* (*1*). Subsequently, 1 strain of RIOMV was isolated from *O. microtis *rats in Peru, designated HTN-007 (*2*); and 2 strains were recovered in the Brazilian Amazon from *O. microtis* rats (RIOMV-3) and uncharacterized species of rodents of the genus *Oligoryzomys* (RIOMV-4) (*3*). Recently, HPS cases associated with RIOMV have been reported: 2 cases in Peru (*4*) and 1 case in French Guiana (caused by a variant named Maripa virus) (*5*). We report isolation of a strain of RIOMV from a patient with fatal HPS in Brazil.

In June 2011, a 28-year-old man was admitted to the Tropical Medicine Foundation Dr. Heitor Vieira Dourado, Amazonas State, with a 4-day febrile illness that included nonproductive cough, myalgia, and headache. Laboratory testing revealed hematocrit within reference range (43.9%), thrombocytopenia (27,000 cells/mm^3^), elevated levels of liver enzymes (alanine transaminase 347 IU/L, aspartate transaminase 139 IU/L), creatinine (1.2 mg/dL), and urea (40 mg/dL). Laboratory testing ruled out malaria, leptospirosis, and dengue. About 24 hours after hospitalization, the patient experienced hypotension, progressive dyspnea, and acute respiratory distress. Thoracic radiographs revealed bilateral diffuse alveolar pulmonary infiltrates. Despite empirical treatment with antimicrobial drugs, mechanical ventilation, and inotropic therapy, the patient’s clinical condition deteriorated and he died on day 6 after illness onset.

The patient, who had no history of travel, resided on a submerged region in the western floodplain of the Solimões-Amazon River, Amazonas, a state with low population density (6.2 persons/square mile), in a rural area of Careiro da Várzea Municipality (3°11′53′′S, 59°52′18′′W), where access is possible only by boat. He had a history of contact with rodents not only at home but also in the boat he used. A serum sample collected on day 6 after illness onset was evaluated for hantavirus by serologic and PCR testing. ELISA result was positive for IgM and IgG against recombinant nucleocapsid protein (N) of the Juquitiba virus (*6*). Viral genome was detected by reverse transcription PCR, and the complete genomic small segment sequence, designated LH60_11/Hu (GenBank accession no. KF584259), was determined (*7*). This sequence was compared with a reference panel of sequences that covered the diversity of most hantaviruses in South America and was subjected to phylogenetic analysis by MrBayes software version 3.1.2 (*8*). Nucleotide and amino acid sequence similarities between all taxa for the partial N gene were calculated by using MegAlign version 5.05 (DNASTAR, Inc.; Madison, WI, USA). The best-fit evolutionary model general time reversible + Γ + proportion invariant was determined by using MEGA version 5.2.2 (http://www.megasoftware.com) of the dataset compiled only 905 nt of the N gene to include sequences of Anajatuba and Rio Mearim viruses from Brazil for comparison.

Bayesian analysis indicated that strain LH60_11/Hu is closely associated with rodent-derived RIOMV-3/Olm strain (Itacoatiara, Amazonas State) and in a sister relationship with RIOMV-4/Olsp strain (Alto Paraíso, Rondônia State) from Brazil (Figure). Analysis of the partial sequence revealed 86.6%–95.4% of genetic identity with the strains recovered from rodents and 83.4% with the Maripa virus strain from humans. The sequence from the human patient in Peru was not available for comparison.

**Figure:**
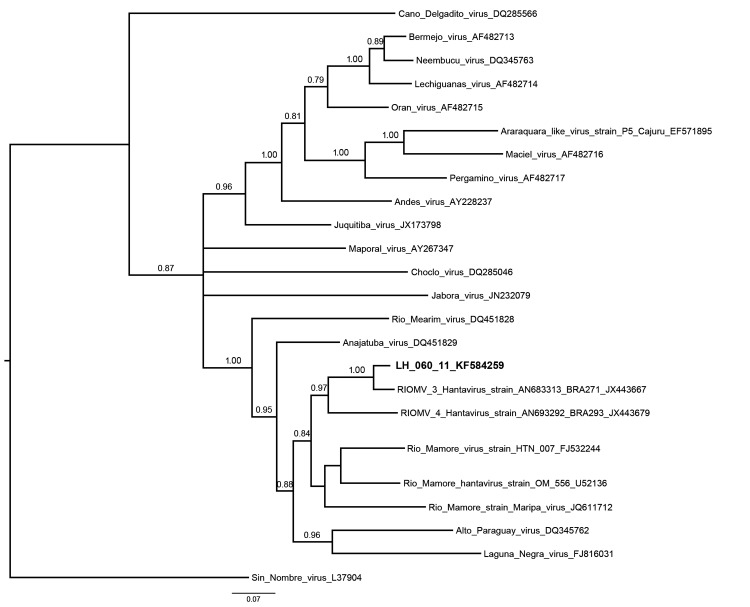
Phylogenetic relationships among hantaviruses were estimated by using the Bayesian Markov chain Monte Carlo method implemented in MrBayes version 3.1.2 (*8*). The relationships were based on the initial 905-nt fragment of the small segment. The numerical value ≥0.7 at the node indicates the posterior probability replicates that supported the interior branch. The branch labels include GenBank accession number and virus species or strain. Boldface indicates the reference sequence; scale bar indicates nucleotide substitutions per site. RIOMV, Rio Mamore virus.

In July, the patient’s house and environment were investigated; accumulation of garbage and other waste in homes that were still flooded was observed. We obtained and tested serum samples from 15 healthy residents (10 female, 5 male) with a recent history of acute fever; IgG against hantavirus was detected in samples from 3 women (17, 25, and 57 years of age).

This case report describes RIOMV as a highly pathogenic agent of HPS in Brazil. The location of the patient with this fulminant case of HPS, Careiro da Várzea, borders the Municipality of Itacoatiara, where RIOM-3–infected *O. microtis* rats and the first HPS case in Amazonas State, with no etiologic identification so far, have been reported (*3*,*9*). Careiro da Várzea is part of an area in which grain production is expanding, an activity that attracts rodents to human dwellings, especially those in lowland regions that are constantly flooded.

The close association between the sequences from the human and the *O. microtis* rat (>98% aa identity) suggests that the patient might have been infected as a consequence of close physical contact with an RIOMV-infected *O. microtis* rat. The geographic distribution of these rats and, thus, the potential area at risk for transmission of RIOMV is vast, including 5 Brazilian states in the Amazon Basin and contiguous lowlands of Peru, Bolivia, and Paraguay (*10*).

This study confirms the notion that ROIMV is a highly pathogenic hantavirus. Recent recognition of RIOMV as a causative agent of HPS might be attributed to either increased awareness by local physicians or improved diagnosis of hantavirus infections. This finding emphasizes the need for extensive molecular investigation of undiagnosed infections because of the shared clinical features with other diseases endemic to this region (e.g., malaria and dengue). 
